# The effect of ketamine and D-cycloserine on the high frequency resting EEG spectrum in humans

**DOI:** 10.1007/s00213-022-06272-9

**Published:** 2022-11-19

**Authors:** J. F. Nottage, A. Gabay, K. De Meyer, K. F. Herrik, J. F. Bastlund, S. R. Christensen, S. Gijsen, M. A. Mehta

**Affiliations:** 1 Department of Psychological Sciences, Birkbeck University of London, London, WC1E 7HX UK; 2 Department of Neuroimaging, Institute of Psychiatry, Psychology and Neuroscience, King’s College London, London, SE5 8AF UK; 3 IXICO, London, UK; 4 H. Lundbeck A/S, Valby, Denmark; 5 Neurocomputation and Neuroimaging Unit, Freie Universität Berlin, Berlin, Germany; 6 Berlin School of Mind and Brain, Humboldt-Universität zu Berlin, Berlin, Germany

**Keywords:** Ketamine, EEG, D-Cycloserine, Antidepressant, Gamma

## Abstract

**Rationale:**

Preclinical studies indicate that high-frequency oscillations, above 100 Hz (HFO:100–170 Hz), are a potential translatable biomarker for pharmacological studies, with the rapid acting antidepressant ketamine increasing both gamma (40–100 Hz) and HFO.

**Objectives:**

To assess the effect of the uncompetitive NMDA antagonist ketamine, and of D-cycloserine (DCS), which acts at the glycine site on NMDA receptors on HFO in humans.

**Methods:**

We carried out a partially double-blind, 4-way crossover study in 24 healthy male volunteers. Each participant received an oral tablet and an intravenous infusion on each of four study days. The oral treatment was either DCS (250 mg or 1000 mg) or placebo. The infusion contained 0.5 mg/kg ketamine or saline placebo. The four study conditions were therefore placebo-placebo, 250 mg DCS-placebo, 1000 mg DCS-placebo, or placebo-ketamine.

**Results:**

Compared with placebo, frontal midline HFO magnitude was increased by ketamine (*p* = 0.00014) and 1000 mg DCS (*p* = 0.013). Frontal gamma magnitude was also increased by both these treatments. However, at a midline parietal location, only HFO were increased by DCS, and not gamma, whilst ketamine increased both gamma and HFO at this location. Ketamine induced psychomimetic effects, as measured by the PSI scale, whereas DCS did not increase the total PSI score. The perceptual distortion subscale scores correlated with the posterior low gamma to frontal high beta ratio.

**Conclusions:**

Our results suggest that, at high doses, a partial NMDA agonist (DCS) has similar effects on fast neural oscillations as an NMDA antagonist (ketamine). As HFO were induced without psychomimetic effects, they may prove a useful drug development target.

**Supplementary Information:**

The online version contains supplementary material available at 10.1007/s00213-022-06272-9.

## Introduction

Electroencephalography (EEG) is a non-invasive, inexpensive, neurophysiological technique which has been demonstrated to be a source of potentially powerful pharmacodynamic biomarkers (PD biomarkers) (Hong et al. 2010; Jobert et al. 2012; Muthukumaraswamy et al. 2013; de la Salle et al. 2016; Grent-'t-Jong et al. 2018). Such EEG PD biomarkers are frequently translatable between experimental animal and human studies (Lazarewicz et al. 2010a, b; Qi et al. 2018). During the early phase of CNS drug development, such a PD biomarker could assist in demonstrating mechanistic efficacy, dose selection and assessing equivalence or difference between compounds. EEG biomarkers are typically of two types; firstly, measures of spontaneous, non-time-locked oscillations capture the background ongoing activity and secondly, event-related potentials (ERPs) quantify the time-locked neural response to an event such as sensory stimulation. This current paper focusses on spontaneous oscillations after decomposition into the frequency-amplitude domain by spectral analysis. Historically, only EEG oscillations below 30 Hz have been considered because higher frequencies are prone to contamination by various non-neural sources of noise. More recently gamma oscillations (up to 100 Hz) have been investigated, with methods for dealing with the noise being developed (Nottage et al. 2013; Nottage and Horder 2015) and successfully applied to human pharmacological data (Nottage et al. 2015). Animal electrophysiology studies show that gamma is not the highest frequency band to be of interest in pharmacological investigations, as recent pre-clinical work has shown clear drug-related effects in high-frequency oscillations (HFO) (100–170 Hz) (Hunt et al. 2006, Hansen, Agerskov et al. 2019, Yan, Suzuki et al. 2022). Furthermore, oscillations in this frequency band also occur in humans as they have been observed in intracranial recordings of people with epilepsy. Although they are more pronounced near to the seizure zone, HFO also occur in healthy occipital, parietal, frontal and temporal regions of cortex (Alkawadri et al. 2014; Guragain et al. 2018) and are associated with brain activity in humans and animals (Crone et al. 2011). However, sometimes gamma and HFO signals may be due to broad band noise from neuronal firing (Ray et al. 2008), although neuronal firing and gamma sometimes dissociate (Leszczyński, Barczak et al. 2020). Furthermore, clear gamma waves have been seen in rats and oscillations below and above 60 Hz can be temporally dissociated (Zheng et al. 2016). It is unclear whether the gamma and HFO signals occurring in the human scalp EEG are broad-band signals or oscillations.

Spontaneous oscillations, measured with EEG in the theta, alpha and beta frequency bands have also been found to be altered in depression (Newson and Thiagarajan 2018), and these frequency bands have been used for investigations into potential treatments for depression (Michel and Pascual-Leone 2020). Recently, there is growing interest in the possibility of using higher frequency bands such as gamma and HFO as biomarkers in the development of antidepressants (Fitzgerald and Watson 2019; Gilbert and Zarate 2020). Here, ketamine is of interest because of the potential effects on gamma oscillation, both acutely (which accompany the psychotomimetic effects) and delayed, or post-acutely (accompanying the antidepressant effects). Acutely, increases in neural gamma oscillations have been consistently shown following ketamine administration in animals and humans (Rivolta et al. 2015; Shaw, Saxena et al. 2015; de la Salle et al. 2016). Whilst ketamine acts in the ion channel of the NMDA tetramer, DCS is a partial agonist at the glycine site on the NMDA receptor complex. There is some evidence from older and more recent studies that DCS—acting on the NMDA receptor through a different mechanism to ketamine—may have some antidepressant effects (Crane 1959; Heresco-Levy et al. 2006; Heresco-Levy et al. 2013; Kantrowitz et al. 2015; Chen et al. 2019), although large-scale trials are needed to confirm this. In animals, drug effects on HFO have also been shown (Hunt et al. 2006, Hansen, Agerskov et al. 2019, Manduca et al. 2020), with both ketamine and a high dose of DCS producing a similar effect on HFO in rodents. However, whilst ketamine also gives rise to a pronounced increase in the gamma band, only a small increase in high gamma (60–100 Hz) was produced by DCS, and only at the highest dose (Hansen, Agerskov et al. 2019).

Alterations in gamma have been argued to be associated with both schizophrenia (Tanaka-Koshiyama et al. 2020; Bianciardi and Uhlhaas 2021; Chung et al. 2022) and drug-induced psychosis (Nottage et al. 2015). Also, in one of our previous studies with Δ-9-tetrahydrocannabinol (THC), we found that the psychomimetic symptoms induced by THC were positively correlated with the ratio of low gamma activity in posterior channels to the magnitude of high beta activity in frontal channels (PLG/FHB ratio) (Nottage et al. 2015) but it is not known whether a similar correlation would be observed with the psychomimetic effects of ketamine.

There are fewer studies of gamma band activity measured in the post-acute phase after ketamine administration, i.e. after ketamine is cleared from the blood (Gilbert and Zarate 2020). In rodents, an increase in gamma band activity after low dose ketamine was not detectable at 90 min (Manduca et al. 2020). In patients with treatment-resistant depression, there is some suggestion that increased gamma band activity 6–7 h post infusion is associated with clinical response (Cornwell et al. 2012) and the magnitude of low gamma (30–50 Hz) oscillations at 6–9 h post-infusion was correlated with HNK metabolite levels after ketamine relative to placebo. These metabolite levels were, subsequently, correlated with poorer clinical outcome (Farmer et al. 2020). None of these studies in humans examined the effects of ketamine on HFO.

The current study had two primary aims; firstly, we wished to assess the feasibility of using frequencies in the 100–170-Hz (HFO) range as translational markers of drug effect in human experimental studies, and secondly to determine the effect of ketamine and DCS on EEG oscillations in higher frequency bands and their associations with psychotomimetic measures. We hypothesised that both D-cycloserine and ketamine would increase frontal HFO as seen in experimental animals (Hunt et al. 2006, Hansen, Agerskov et al. 2019, Manduca et al. 2020), but that DCS would not produce detectable psychotomimetic effects. This will lend support to the hypothesis that modulation of NMDA receptors via the glycine site produces electrophysiologically similar effects to pore block for high-frequency EEG. This could thus be a viable approach to reduce NMDA receptor activity without producing psychotomimetic effects whilst affecting EEG signals similarly. We used single doses of 250 mg and 1000 mg DCS in order to capture the predicted range of potential effectiveness of DCS in patients (Heresco-Levy et al. 2013). We also hypothesised that there would be an association between psychotomimetic scores and the high-frequency EEG measures, particularly the posterior low gamma to frontal high beta ratio as described above. In addition to collecting resting-state EEG for the pre-defined primary outcome measure (frontal midline resting HFO amplitudes), EEG data for secondary outcome measures comprising electrophysiological responses in four event-related paradigms were also acquired (Auditory Deviance, Auditory Steady State Response, Button Pressing and Backward Counting). The analysis of these secondary EEG outcomes will be reported separately. Finally, we collected EEG data after the acute psychotomimetic experiences of ketamine had passed to explore the prediction that the HFO response will no longer be present.

## Methods

### Study design

This was a partially double-blind, single cohort 4-way crossover, comparator, ‘double-dummy’ placebo-controlled and counterbalanced study in healthy male volunteers. Each participant attended four study days. On each day, they received an oral tablet consisting of 250 mg or 1000 mg of DCS, or, on 2 days, placebo. Two hours 30 min after oral dosing, which was the expected time of the maximum serum concentration of DCS, they received an intravenous infusion. This was saline placebo on three of the days, but on one of the days in which the oral dose was placebo, the infusion contained 0.5 mg/kg ketamine. The four study conditions were therefore as follows: placebo-placebo, 250 mg DCS-placebo, 1000 mg DCS-placebo or placebo-ketamine. Study assessments were made prior to drug administrations, during the ketamine infusion/peak DCS effect to capture the acute drug effect and 2 h after the infusion to capture a time when antidepressant effects of ketamine are detectable. A flow diagram of the study protocol is included in Online Resources 1, Fig. ES1.

This study was approved by the King’s College London Research Ethics Committee (reference: HR-16/17–4164) and the Health Research Authority and conducted in the Clinical Research Facility, King’s College Hospital, London.

### Participants

Twenty-seven participants were recruited to the study. Three participants were withdrawn from the study. (One had a positive urine drug screen on one of the visits, the second had a fever outside of the study visits and commenced an herbal treatment and the third had food poisoning outside of the study visits and once recovered we had difficulties with rescheduling.) and their data was excluded from further analyses. Hence, 24 participants contributed data that were analysed, with 6 participants following each of the 4 drug sequences. The mean time between study periods was 8.26 days (standard deviation 4.0 days). Most study sessions were 7 days apart, and only one session across all subjects, was less than this, at 6 days. All participants were right-handed male volunteers, and the age range was 19–38 years (mean = 26). The study only included male participants to avoid the complication of effects of the menstrual cycle on EEG oscillations in females (for example see de Souza et al. 2022).

Inclusion criteria required normal ECG, standard laboratory blood screens and urinalysis, and alcohol consumption within the recommended guidelines at the time of the study (< 28 units per week). Exclusion criteria included a history of neurological or psychiatric illness, physical illness, and positive urine drug test on the screening or study days.

Participants were randomised into the study and assigned to a sequence of treatment administrations by means of a computer-generated, pseudo random code using a Williams square which is a generalised Latin square that controls for order and first-order carryover effects (Williams 1949). The study was partially double-blind; for ketamine administration, the study clinician and research nurse were not blind to ketamine, but the participants and the researchers who acquired and analysed the data were blinded. Different researchers carried out the acquisition and analysis stages.

### Drugs

Ketamine was administered as an infusion at a rate of 0.5 mg/kg over 40 min. The racemic ketamine dose (0.5 mg/kg) and route of administration (IV) were selected based on literature as well as experience from previous studies in order to mimic the infusion profile typically used to produce antidepressant effects whilst minimising any potential side effects (McCloud et al. 2015). DCS was administered orally at 250 mg and 1000 mg. The study included saline placebo infusion for ketamine, and oral (lactose) placebo for DCS. To eliminate carryover effects between study periods, there was at least a 7-day washout period between study days.

In the fed state, plasma D-cycloserine levels peak around 2.5–3 h (Baron et al. 1955; Zítková and Tousek 1974; Zhu et al. 2001) with a half-life of 15–25 h (Zítková and Tousek 1974). The central bioavailability is excellent (Baron et al. 1955), with peak CSF levels occurring at 2 h (Baron et al. 1955) corresponding to at least 79% of peak serum levels (Baron et al. 1955; Holdiness 1985).

### Data acquisition

#### Electroencephalography (EEG)

A Compumedics Neuroscan Synamps2 amplifier was used with a sampling rate of 10 kHz. Filter settings were 0.01-Hz low-pass filter and 2-kHz high-pass filter. A 64-channel cap was used, arranged in the 10–10 system, with the left Mastoid acting as the recording reference and with the ground located at POz. Additional electrodes were located above and below the right eye, at the left and right canthus and on the nose.

EEG was acquired at three timepoints: Time 1 (T1 or baseline) was before oral dosing and infusion; Time 2 (T2—during the plasma infusion) begun 15 min after the start of the infusion; Time 3 (T3—‘post-infusion’) was 2 h after the start of the 40-min infusion. T2 was chosen to capture the effect of ketamine and commenced 15 min after infusion commenced to ensure sufficient ketamine exposure after the likelihood of immediate side effects had passed. T3 was chosen as the time when antidepressant effects can be detected in depression trials, after initial subjective effects had passed.

#### Resting EEG

Each recording session lasted 4 min and was executed with eyes closed in a reclining position. Every minute, participants were required to open their eyes for 10 s, and then close them again, in response to computer-generated auditory commands. This was to ensure that participants remained awake. Our primary research question for this study was whether frontal HFO are altered by ketamine and DCS. When the eyes are open, there is contamination in the frontal EEG signal from the extra-ocular muscles, the orbicularis oculi muscles (during blinks), as well as the levator palpebrae superioris muscles and frontalis muscles (when holding the eye-lids up and looking upwards). In order to minimise these sources of noise, only the eyes-closed data was included in the spectral analysis reported here. To ensure that participants were correctly following the instructions to open and close their eyes, the signal from electrodes above and below the eyes (VEOG) and from the outer canthi of both eyes (HEOG) were recorded, and a trigger was sent to the EEG at the time of the automated verbal instruction to open or close the eyes. When the eyes are opened or closed, there is a voltage shift, and with the eyes closed, there should be no blinks or saccadic activity. The researcher who was recording the EEG was trained to monitor carefully that the signals from the VEOG and HEOG electrodes showed compliance with the instructions.

#### Drug plasma levels

Two blood samples were collected and assayed for levels of ketamine and its metabolites (norketamine, 2S-6S-hydroxynorketamine and 2R-6R-hydroxynorketamine): at 3h15 and at 4h30 after tablet dosing. For D-cycloserine plasma levels, 3 blood samples were collected: at 2h15, 3h30 and 5h15 post tablet administration.

#### Symptom scores

The PSI (Psychotomimetic States Inventory) (Mason et al. 2008) was used to measure psychotomimetic effects of the drugs. This inventory is sensitive to the effects of ketamine (Mason et al. 2008) and shows excellent test–retest reliability for these effects (De Simoni et al. 2013). The PSI has six subscales: delusional thinking, perceptual distortion, cognitive disorganisation, anhedonia, mania, paranoia. Questionnaires were administered at baseline (before oral dosing or infusion), during the infusion, at the end of infusion, and 4 h after the start of the infusion.

### Data analysis

EEG signal processing and spectral analysis was performed in MATLAB (9.3.0). Data was imported from NeuroScan with EEGLAB (14.2.1) (Delorme and Makeig 2004), and then pre-processed using GammART (a MATLAB toolbox, developed at King’s College London, for analysing high-frequency EEG data). Low-frequency noise was reduced using a 1-Hz high-pass filter, followed by powerline noise reduction using noise cancellation (Nottage et al. 2013). Further details of the analysis process are included in Online Resources 4. The EEG data was visually inspected to ensure that there were no unusual artefacts and to check that the VEOG and HEOG signals showed that the eyes were being opened and closed correctly.

The EEG was then re-referenced to Cz as midline channel locations, such as Fz, Cz and Pz, have been shown to have minimal EMG contamination (Whitham et al. 2008; Nottage et al. 2013; Volker et al. 2018). This is because the muscle fibres for the frontalis, temporalis and neck muscles are located only in peripheral locations, and not under these central electrodes. Therefore, we used bipolar channels utilising only the data from Fz-Cz and Pz-Cz. However, gross, widespread, EMG artefacts originating in peripheral sites, such as the jaw clenching contraction of the masseter muscles, frowning activation of the Frontalis muscles or EMG from neck movements, can contaminate even midline channels (Goncharova, McFarland et al. 2003). Any EEG segments containing such gross artefacts were rejected with a method adapted from Fieldtrip’s ‘ft_artifact_muscle’ function (Oostenveld et al. 2011) using EEG data for 14 peripheral channels which can be strongly affected by this kind of noise (FP1,FP2,F7,F8,FT7,FT8,T7,T8,TP7,TP8,P7,P8,O1,O2). Visual inspection of experiments confirmed that this methodology conservatively labelled high-amplitude EMG artefacts in peripheral channels which could affect central channels. The maximum rejection in a single session was 43%. Rejection rates were similar across conditions; at Time 2, the rejection rates and standard deviations were as follows: placebo: 6.4% (std: 11.1%), ketamine: 5.6% (std: 8.6%), 1000 mg DCS: 6.9% (std: 7.6%), 250 mg DCS 5.7% (std: 9.7%). Two EEG recordings from one participant had to be rejected due to excessive, continuous, orbicularis oculi contractions which contaminated Fz. Data from faulty electrodes in one session each for two participants were also rejected. All other data was submitted for analysis.

Moving-window, short-time Fourier transforms with a Hann window were applied to the eyes-closed data. For the Fourier transforms, HFO were quantified using 100-ms windows, gamma using 200-ms windows and beta using 500-ms windows, with a 2.5% shift between consecutive windows. The pre-defined primary outcome measure for this study was the magnitude of resting frontal midline HFO, and for this we used the Fz channel, referenced to Cz (Fz-Cz). For comparison, data from a midline posterior channel (Pz-Cz) was also analysed. The data was averaged over 5 high-frequency bands. Whilst standardised frequency bands for pharmacological studies have been recommended for lower frequencies, the frequency bands for higher frequencies are still tentative (Jobert et al. 2012). For the current study, the frequency bands were chosen based on two previous pieces of research; firstly, a study with DCS and ketamine in rodents showed that there could be peaks in the high-frequency oscillations HFO:100–170 Hz, high gamma (HG): 65–100 Hz and mid-gamma (MG): 40-65 Hz, which could react differently to the drugs (Hansen, Agerskov et al. 2019). We also previously found that spectral amplitudes above and below 27 Hz, in the human EEG, responded in opposite directions to THC (Nottage et al. 2015), so we defined low gamma (LG) as 27–40 Hz and high beta (HB) as 20–27 Hz. The PLG/FHB ratio was calculated using LG at Pz-Cz divided by HB at Fz-Cz. To ensure normality, EEG measures were either used as a ratio, a percentage change or else log-transformed for analysis. Although we analysed a range of frequencies above 20 Hz, frontal HFO was our primary measure of interest.

Statistical analysis was carried out with IBM SPSS. For each drug and measure separately, the data was analysed using mixed models analysis at each timepoint. Condition was included as a fixed effect, with 3 levels for the DCS analysis (1000 mg, 250 mg and placebo) and two levels for the ketamine analysis (drug and placebo). Day was included as a covariate, whilst subject was included as a random effect. For EEG measures, a linear mixed model was used, whilst for the PSI scores, a generalised mixed model was used utilising multinomial logistic regression. Friedman’s test was used for post hoc tests on individual PSI subscales.

Pearson’s correlation coefficients were calculated between the mean ketamine, norketamine, HNK and DCS plasma levels and the amplitude in each frequency band which showed a significant change with drug. For associations with symptoms, Spearman’s Rho was calculated.

## Results

### Resting-state EEG at T2 (ketamine exposure peak)

#### High-frequency oscillations (100–170 Hz)

**Fig. 1:**
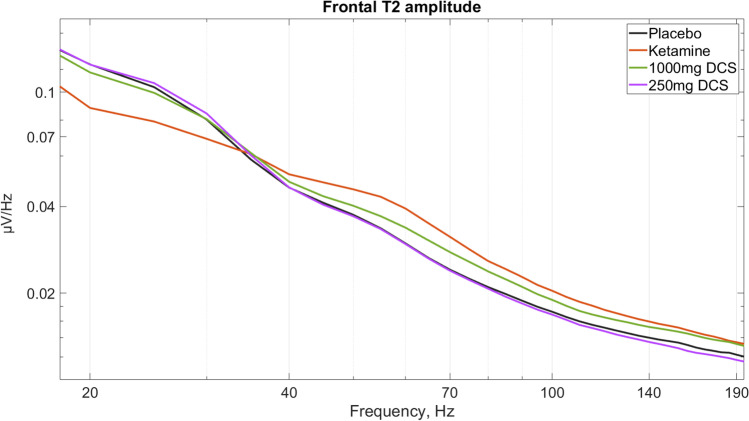
Frontal EEG amplitudes during infusion at T2 (peak of ketamine exposure), showing the significantly increased spectral amplitudes above 40 Hz induced by ketamine and 1000 mg DCS

The primary aim of this study was to investigate the effect of DCS and ketamine on midline frontal HFO amplitude. As expected, we did not observe any significant frontal HFO differences at baseline (T1) before drug administration. However, we found that, at T2—the time of the peak drug plasma concentrations (during the ketamine or saline infusion), there was a main effect of condition for both drugs compared with placebo (see Fig. 1). Frontal HFO magnitude was increased both by ketamine (placebo = 0.643 µV, ketamine = 0.773 µV, *F*(1,44.9) = 21.27, *p* = 0.00014*, effect size = 0.63) and by DCS (*F*(2,44.8) = 4.75, *p* = 0.013*). In pairwise comparisons of fixed effects for DCS, the 1000-mg dose of DCS significantly increased the amplitude of frontal midline HFO compared with placebo (1000 mg DCS: 0.751 µV, *p* = 0.010*, effect size = 0.60), but the 250-mg dose did not produce a significant change. (* significant when corrected for multiple comparisons). Also, 1000 mg DCS significantly increased the magnitude of frontal HFO compared to the 250-mg dose of DCS (250 mg DCS: 0.645 µV, *p* = 0.012). We also carried out a mixed model analysis with only ketamine and 1000 mg DCS, to test whether the HFO magnitude differed between these two conditions and found that there was no main effect of drug condition (*p* = 0.62) in this comparison.

**Fig. 2:**
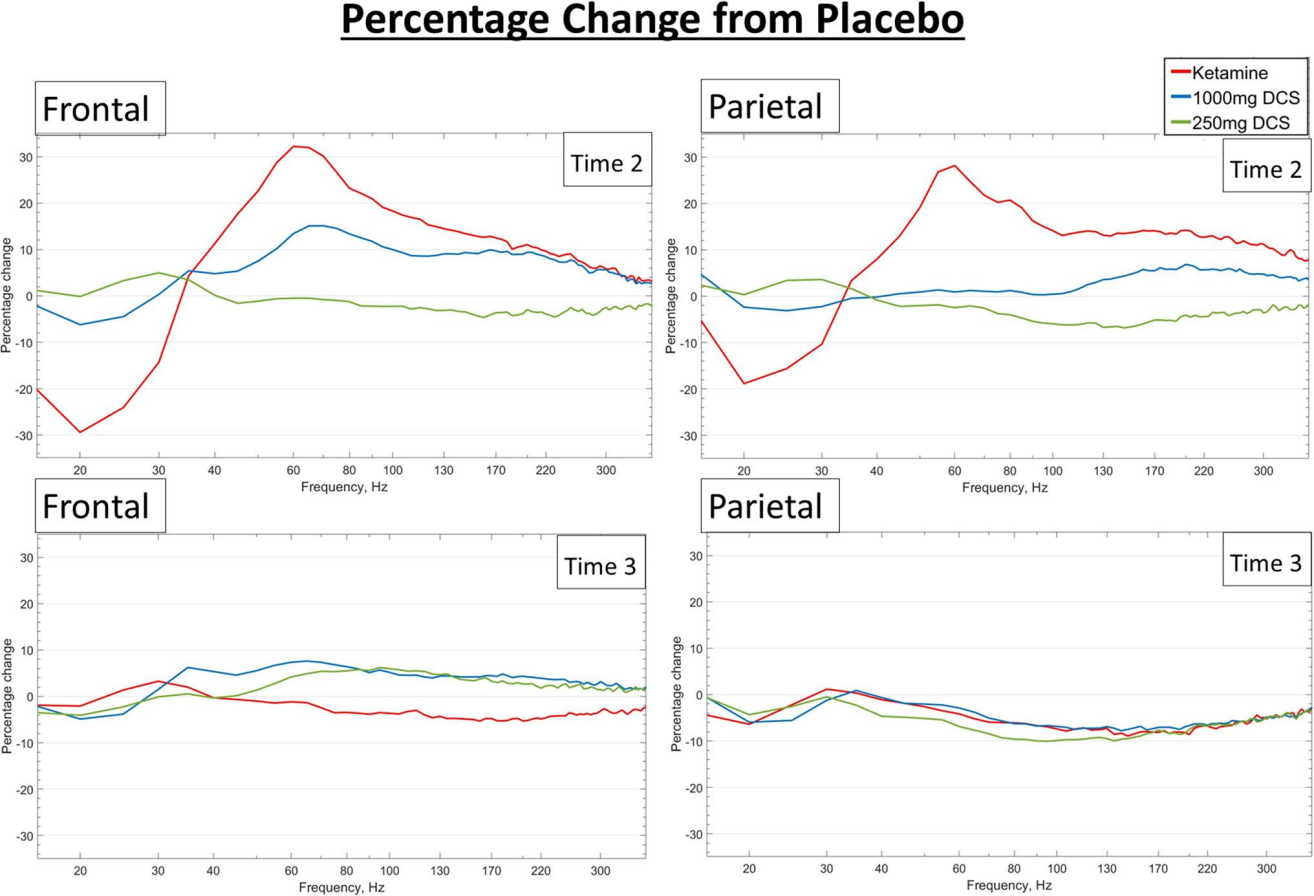
Percentage change in EEG amplitudes from placebo at T2 (peak) and T3 (post peak of ketamine exposure) for ketamine and DCS. There were significant changes for ketamine and DCS at time T2, but no significant changes at T3

Having established that both ketamine and 1000 mg DCS increased HFO in the frontal location, which was the a priori region of interest, we tested if these HFO effects were consistent for a posterior location (Pz-Cz). Compared with placebo, there was a significant main effect of condition at this parietal location for both ketamine (see Fig. 2) and DCS (*F*(2,44.2) = 4.13, *p* = 0.023). For DCS, this consisted of an increase in amplitude with 1000 mg DCS compared with placebo (1000 mg DCS: 0.760 µV, placebo: 0.654 µV, *p* = 0.011) (see Fig. 2). Also, as with the frontal location, the parietal HFO magnitudes did not differ significantly between the ketamine and the 1000 mg DCS sessions (*p* = 0.76), and 250 mg DCS did not differ significantly from placebo.

**Fig. 3:**
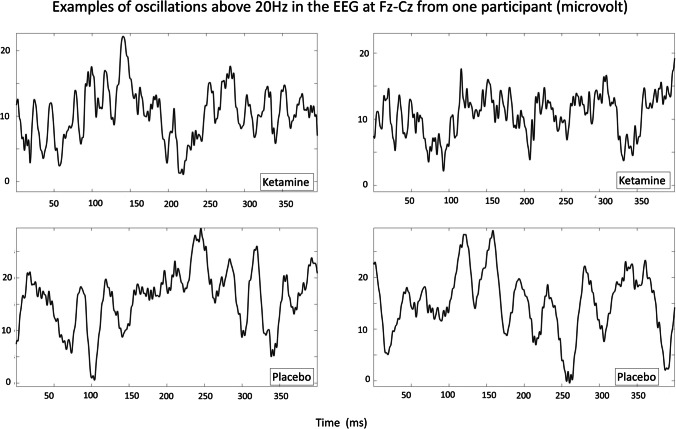
Four examples of the EEG signal at Fz, referenced to Cz from a single participant. These show oscillations above 20 Hz in the ketamine (top line) and placebo (bottom line) condition at T2. In the placebo condition, the signal is dominated by beta, with only a small amount of gamma, whereas gamma and HFO dominate under ketamine. A low-pass FIR filter, with a cut-off of 300 Hz, has been applied to the data to remove amplifier noise for visualisation purposes. This filter was not applied to the data used for spectral analysis

In some cases, gamma and high-frequency oscillations could be observed in the EEG signal, such as in the examples shown in Fig. 3.

#### Gamma and high beta (20–100 Hz)

In the comparison between ketamine and placebo, there was a significant main effect of drug condition on the frontal midline amplitude of MG, HG and HB, but not LG, with large or very large effect sizes (see Table 1 for details). Ketamine significantly increased the amplitude of MG and HG, and decreased the amplitude of HB, compared with placebo. In the DCS analysis, there was a significant main effect of condition on midline frontal MG (*F*(45.0, 2) = 9.42, *p* = 0.00038) and HG (*F*(44.9, 2) = 9.77, *p* = 0.00030) but not LG or HB (see Fig. 1 and Fig. 2). In the pairwise comparisons, MG was significantly increased by 1000 mg DCS compared with placebo (1000 mg DCS: 1.066 µV, placebo: 0.954 µV, *p* = 0.00052), as was HG (1000 mg DCS: 0.792 µV, placebo: 0.664 µV, *p* = 0.00034) but not HB or LG. Also compared to the 250-mg dose of DCS, 1000 mg DCS significantly increased the magnitude of MG (0.953 µV, *p* = 0.00046) and HG (0.668 µV, *p* = 0.00047). In the comparison between ketamine and 1000 mg DCS, the amplitudes for ketamine were significantly higher for frontal MG (*F*(1, 22.14) = 11.63, *p* = 0.0025), but not HG (*p* = 0.095).

**Table 1:** Frontal amplitudes (Fz-Cz)—T2 (peak) ketamine vs placebo

	*High beta* *(20–27 Hz)*	*Low gamma* *(27–40 Hz)*	*Mid-gamma* *(40–65 Hz)*	*High gamma* *(65–100 Hz)*	*HFO* *(100–170 Hz)*
*F*	**35.1**	4.88	**47.1**	**42.9**	**21.27**
*p*	**6 × 10**^**−6**^	0.038	**6 × 10**^**−7**^	**1 × 10**^**−6**^	**0.00014**
*df*	**1, 22.2**	1, 22.0	**1, 22.3**	**1, 21.8**	**1, 22.0**
*Cohen’s d*	** − 1.06**	** − **0.34	**1.05**	**1.06**	**0.63**
*Ketamine (µV)*	**1.126**	1.190	**1.202**	**0.860**	**0.773**
*Placebo (µV)*	**1.523**	1.288	**0.625**	**0.664**	**0.643**

Significant effects, after Bonferroni correction for multiple comparisons, are shown in bold. The uncorrected *p* values are shown in the table

The parietal results for ketamine vs placebo were similar to the frontal effects, as can be seen in Table 2. However, even without correction for multiple comparisons, across DCS doses, the parietal effects were not significant for MG or HG at Time 2 (*p* = 0.191 for MG, *p* = 0.153 for HG), although in the pairwise comparisons, the trend was towards an increase in both bands with 1000 mg DCS compared with placebo (MG: *p* = 0.089, HG: *p* = 0.077). As with the frontal location, ketamine and 1000 mg DCS only produced significantly different effects in MG (*F*(1,22.0) = 9.85, *p* = 0.0048), but not HG (*p* = 0.063).

**Table 2:** Parietal amplitudes (Pz-Cz)—T2 (peak) ketamine vs placebo

	*High beta* *(20–27 Hz)*	*Low gamma* *(27–40 Hz)*	*Mid-gamma* *(40–65 Hz)*	*High gamma* *(65–100 Hz)*	*HFO* *(100–170 Hz)*
*F*	**19.1**	3.5	**45.1**	**38.1**	**24.9**
*p*	**0.0002**	0.075	**9 × 10**^**−7**^	**3 × 10**^**−6**^	**5 × 10**^**−5**^
*df*	**1, 21.9**	1, 22.0	**1, 22.1**	**1, 22.1**	**1, 22.1**
*Cohen’s d*	** − 0.61**	** − **0.23	**0.72**	**0.71**	**0.53**
*Ketamine (µV)*	**1.315**	1.196	**1.096**	**0.794**	**0.774**
*Placebo (µV)*	**1.626**	1.285	**0.908**	**0.649**	**0.654**

Significant effects, after Bonferroni correction for multiple comparisons, are shown in bold. The uncorrected *p* values are shown in the table

We also tested the effect of ketamine and DCS, compared with placebo, on the PLG/FHB ratio, and found a significant main effect of condition for ketamine (*F*(1,22.1) = 20.7, *p* = 0.00015). However, the main effect of condition did not reach significance across DCS doses (*p* = 0.075), but in the pairwise test between 1000 mg DCS and placebo, the PLG/FHB ratio was significantly greater with 1000 mg DCS, but only before correction for multiple comparisons (*p* = 0.024).

### Resting-state EEG at T3 (post exposure peak)

In the final EEG session (T3), there was no significant drug effect at any frequency or location for the comparison between ketamine and placebo. For the DCS condition, there were some effects which were significant without correction for multiple comparisons. Specifically, post-peak, there was a main effect of drug on frontal MG (*F*(43.4,2) = 7.11, *p* = 0.0021), HG (F(43.6,2) = 4.67, *p* = 0.015) and HFO (*F*(43.0,2) = 3.69, *p* = 0.033) (see Fig. 2).

In pairwise comparisons of fixed effects, compared to placebo, 1000-mg dose of DCS increased the amplitude of frontal MG (1000DCS: 0.971 µV, placebo: 0.916 µV, *p* = 0.031), HG (1000DCS: 0.736 µV, placebo: 0.634 µV, *p* = 0.0053) and HFO (1000DCS:0.700 µV, placebo: 0.608 µV, *p* = 0.015). Also, with the 250-mg dose of DCS, MG (250DCS: 0.975 µV, *p* = 0.030), HG (250DCS: 0.711 µV, *p* = 0.028) and HFO (250DCS: 0.687 µV, *p* = 0.035) were all increased in magnitude compared with placebo. However, none of these T3 effects survived Bonferroni correction for multiple comparisons. There were no significant changes in the parietal gamma or HFO with DCS.

### Frequencies below 20 Hz

Frequencies below 20 Hz were not part of the a priori outcome measures, but we have included the analysis of peak (T2) low frequencies in the Online Resources 2, for completeness. Ketamine reduced theta magnitude, especially in the 4–8-Hz range in the parietal channel ((*F*(21.5,1) = 20.33, *p* = 0.00018) and also 13–20-Hz magnitude, especially frontally (*F*(21.8,1) = 50.1, *p* = 4 × 10^−7^) (see Tables ES1 and ES2, *p* values not Bonferroni corrected). Also, 1000 mg DCS tended to reduce the frontal 13–20-Hz band and increase in the parietal 10–13-Hz and 8–10-Hz bands compared with placebo.

### Psychomimetic effects of ketamine and DCS measured with PSI at peak

**Fig. 4:**
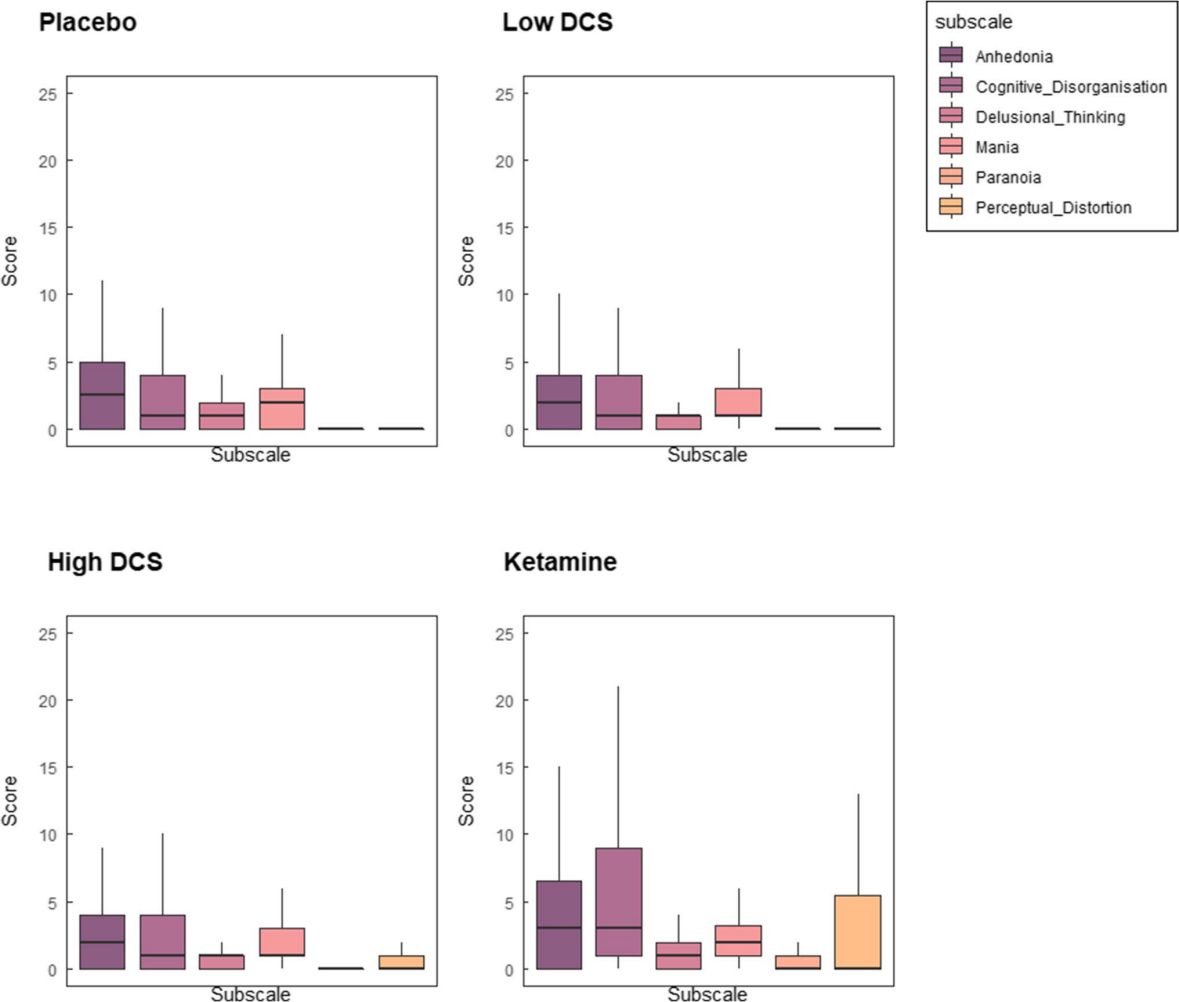
PSI scores: ketamine vs. placebo during infusion

For the comparison between ketamine and placebo, there was a significant main effect of treatment (*F*(1,232) = 108.8, *p* < 0.001, total PSI scores ketamine 39.5, placebo 12.4). Subscale (*F*(5,232) = 21.7, *p* < 0.001), and subscale × treatment interaction (*F*(5,232) = 7.01, *p* < 0.001) and day (*F*(3,232) = 2.916, *p* = 0.035) (see Fig. 4). Friedman’s tests on the individual sub-scale scores show that whilst all subscale scores were increased by ketamine, the largest effect sizes were for perceptual distortion and cognitive disorganisation (see Table 3). DCS did not produce significant changes in PSI scales for either dose when compared individually with placebo (1000 mg DCS: *p* = 0.223, 250 mg DCS; *p* = 0.394).

**Table 3:** PSI scores

	*N*	Mean PSI scores (SD)				Statistical comparison of ketamine with placebo		
		Ketamine	Placebo	1000 mg DCS	250 mg DCS	Test statistic, *Q*	Bonferroni-corrected *p* value	Cohen’s *d*
Anhedonia	22	6.50 (5.1)	3.43 (3.2)	3.09 (2.5)	3.24 (3.0)	9.8	0.01*	0.7
Cognitive disorganisation	22	14.41 (7.2)	4.04 (4.9)	3.91 (4.6)	3.05 (3.7)	16.2	< 0.005**	1.7
Delusional thinking	22	3.55 (3.5)	1.13 (1.3)	1.00 (1.0)	0.81 (0.9)	14.4	< 0.005**	0.9
Perceptual distortion	22	7.77 (4.4)	0.96 (2.12)	1.18 (1.6)	0.48 (0.9)	19.0	< 0.005**	1.9
Mania	21	5.67 (4.0)	2.35 (2.21)	2.45 (2.5)	1.95 (1.7)	8	0.03*	1.0
Paranoia	22	1.82 (2.7)	0.52 (0.99)	0.55 (1.4)	0.19 (0.4)	7.4	0.04*	0.6

For DCS, the effect of condition on total PSI scores was not significant (*F*(2,365) = 1.982, *p* = 0.139). We note that perceptual distortion scores were higher after 1000 mg DCS, although this was not significant after multiple comparisons correction (Friedman’s test: HDCS = 1.18 (1.6), *Q* = 4.5, *p* = 0.034, uncorrected).

### EEG measures and PSI subscales

At T2 (peak), there was no significant correlation between any frequency above 20 Hz and total PSI scores, either using the absolute values or changes from baseline. The correlation coefficients with PSI subscales are shown in the Supplementary Material. As the perceptual distortion subscale showed not only the strongest effect with ketamine, but also a trend towards increased perceptual distortion scores with the high dose of DCS, we looked at correlations with gamma and HFO for this subscale.

For the PLG/FHB ratio, we tested the correlation with the perceptual distortion as it is a measure of the psychosis-like effects of the drugs. Using Spearman’s Rho, the PLG/FHB was positively correlated across all the peak (Time 2) sessions for all conditions with the perceptual distortion sub-scale (*r* = 0.448, *p* = 0.00001). This correlation was still statistically significant when only the Ketamine peak sessions were considered (*r* = 0.450, *p* = 0.036).

### Plasma concentrations

**Fig. 5:**
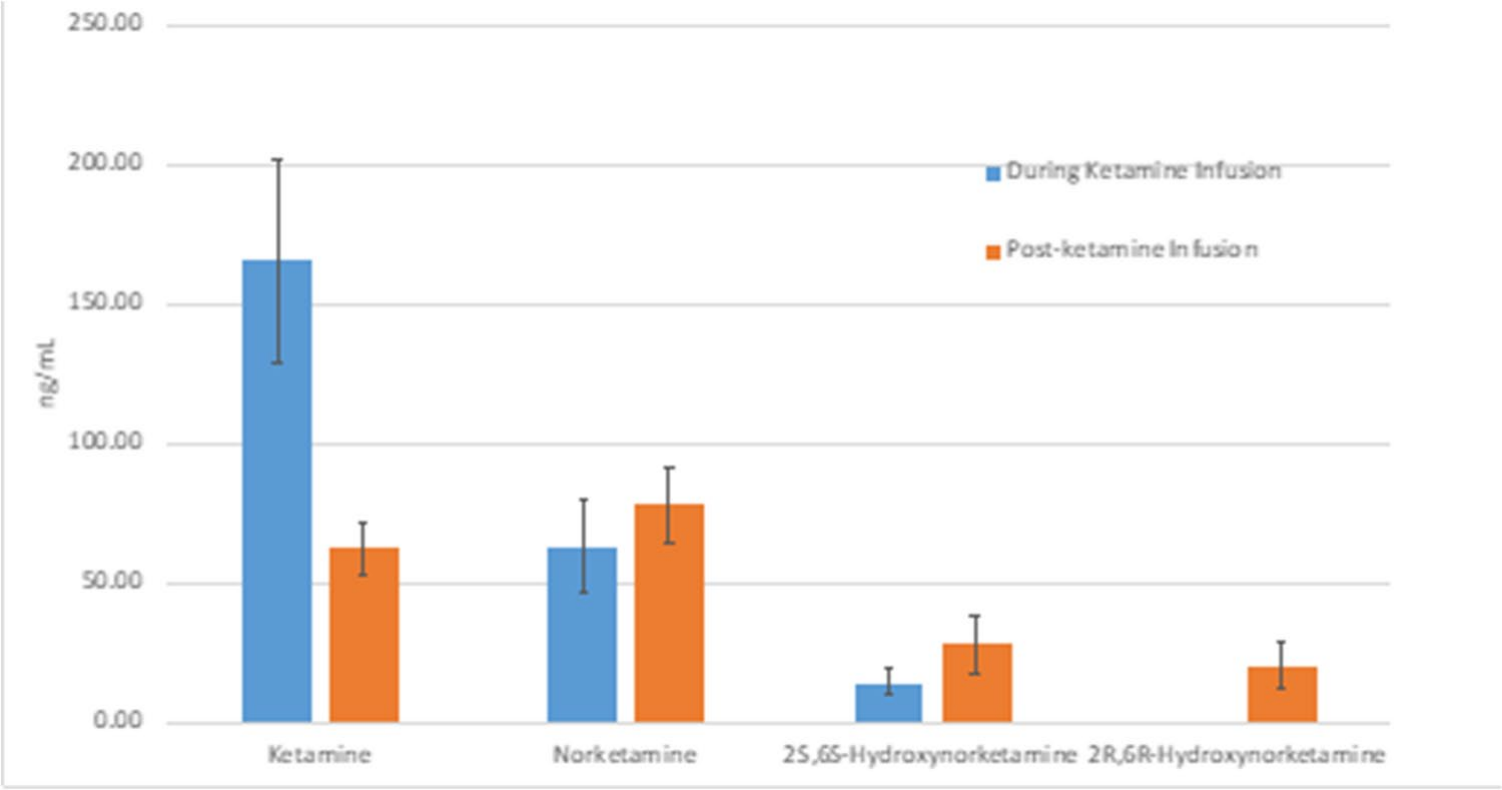
Plasma levels of ketamine and the main metabolites at T2 (peak of ketamine exposure) and T3 (post peak of ketamine exposure). The error bars show standard errors. The bar for 2S, 6S-hydroxynorketamine is omitted because only 4 participants had data available

The plasma levels of ketamine and the main metabolites of interest, norketamine and 2(R,S), 6(R,S)-hydroxynorketamine (HNK) are shown in Fig. 5. For the 2S, 6S-hydroxynorketamine, the samples from four participants were below the level of quantification and were thus excluded. For the (2R, 6R)-hydroxynorketamine, 20 (of 24) participants were below the level of quantification during the infusion so this data is not shown on the histogram and was not included in further analysis. No samples were excluded for the third timepoint.

### EEG correlations with metabolites

Using Pearson’s correlation coefficient, there was no correlation between ketamine plasma levels and the absolute magnitudes or percentage change from placebo (see Online Resource 3, Table ES3) at any timepoint for frequencies above 27 Hz. For norketamine, the percentage increase from placebo of gamma (40–65 Hz), at peak (T2), was weakly associated with the mean norketamine plasma levels (*r* = 0.436, *p* = 0.038), although this did not survive correction for multiple comparisons, and the absolute values were not significantly correlated (*r* = 0.317, *p* = 0.141). In post hoc tests, we tried to match as closely as possible to the methods used by Farmer et al. (Farmer et al. 2020) by combining the post-peak (T3) 27–40-Hz and 40–65-Hz bands (they used 30–50 Hz) and comparing the EEG for the last recording with the maximum plasma concentrations of norketamine and HNK. We did not observe a significant correlation between the post-peak (T3) magnitude of the 27–65-Hz band and either norketamine (*r* = 0.245, *p* = 0.260) or the maximum combined (2S, 6S)-HNK and (2R, 6R)-HNK levels (*r* = 0.055, *p* = 0.82).

As both frontal beta (13–27 Hz) and theta (4–8 Hz) were reduced by ketamine, we tested for correlations of magnitudes of low-frequency bands with ketamine and its metabolites (see Online Resource 3, Table ES4). We observed no associations for theta, but positive correlations with the frontal peak 13–27-Hz magnitude and maximum norketamine, and a negative correlation between peak beta and (2S, 6S)-HNK during the ketamine infusion, as well as with the maximum (2S, 6S)-HNK and (2R, 6R)-HNK levels, measured 2 ½ h later. However, we noticed that this negative correlation with absolute magnitude extended to all frequencies between 8 and 27 Hz, and all EEG timepoints, but was not present in the percentage change from placebo. In fact, when we averaged frontal baseline T1 (pre-treatment) magnitudes across all four study days, these mean values were also strongly negatively correlated with the plasma concentrations of (2S, 6S)-HNK induced during the ketamine infusion at all frequencies between 8 and 20 Hz (8–10 Hz: *r* =  − 0.586, *p* = 0.0066, 10–13 Hz, *r* =  − 0.646, *p* = 0.0021, 13–20 Hz: *r* =  − 0.601, *p* = 0.0050, 20–27 Hz; *r* =  − 0.432, *p* = 0.057). This shows that in our sample, those individuals who generally had high amplitude alpha and beta oscillations in their EEG tended to metabolise ketamine into (2S, 6S)-HNK less rapidly.

## Discussion

In this study, we demonstrated that the data from experimental animals showing increased high-frequency neural oscillations with DCS and subanaesthetic doses of ketamine are translatable to humans. Similar to the findings in rodents (Hansen, Agerskov et al. 2019), we showed that midline frontal HFO magnitude is increased both by ketamine and by DCS. At peak drug concentration, the effect of ketamine and the high dose of DCS on HFO magnitude were indistinguishable at both the frontal and parietal locations. We also found that DCS did not increase gamma and reduce high beta frequencies to the same extent as ketamine. In particular, the parietal 40–65-Hz band, which was dramatically increased by ketamine, was unaffected by either dose of DCS (see Fig. 1), whilst the frontal 40–65-Hz magnitude was increased significantly more by ketamine than the high dose of DCS. The increase in HFO oscillations was transient with ketamine, but with DCS the frontal enhancement appeared more sustained, in line with pharmacokinetics of the oral compound.

### Resting state spectral amplitudes

Our gamma band resting state results are consistent with previous human studies (Gilbert and Zarate 2020), which showed increases in gamma amplitude induced by ketamine, compared to placebo. In an MEG study, S-ketamine (0.006 mg/Kg) was reported to increase 30–90-Hz activity in subcortical (thalamus and hippocampus) and cortical (frontal and temporal cortex) regions, whilst reductions in beta-band power were observed in the precuneus, cerebellum, anterior cingulate, temporal and visual cortex (Rivolta et al. 2015). Another MEG study investigated the effect of 0.5 mg/kg intravenous ketamine on task-related oscillations in visual and motor cortices. Ketamine increased beta amplitude, decreased peak gamma frequency in visual cortex and significantly amplified gamma-band amplitudes in motor and visual cortices (Shaw, Saxena et al. 2015). The two other studies of resting-state EEG with ketamine reported a widespread increase in gamma in scalp electrodes. A current source density analysis in one study showed increased gamma amplitude in the ventromedial prefrontal cortex, both the anterior and posterior cingulate cortex and the anterior insular (de la Salle et al. 2016). In the other study, it was found that ketamine increased gamma connectivity in a network involving occipital, midline and frontal regions (Curic, Andreou et al. 2021). We are not aware of any previous EEG or MEG human investigations of frequencies above 90 Hz with ketamine or of any studies of the gamma band or above with DCS.

The difference between the gamma and HFO DCS effects in the parietal channels in our study point to gamma and HFO not originating from broadband noise in this case. Also, ketamine-induced gamma and HFO were sometimes visible as oscillations in the EEG signal as can be seen in Fig. 3. It is possible for low-pass filters to make spurious oscillations appear when applied to broadband noise. However, these waves occur just below the cut-off frequency, but the cut-off frequency of the low-pass filter applied to the data shown (for visualisation purposes only) was 300 Hz. Therefore, the observed ketamine-induced oscillations, which were around 40–130 Hz in the examples in Fig. 3, could not have been due to the filtering applied.

### Mechanisms of antidepressant action of ketamine and DCS

The exact mechanism of action by which ketamine produces antidepressant effects is not yet clear, but various hypotheses have been proposed (Zanos and Gould 2018; Lavender et al. 2020). The leading hypotheses state that the blockade of NMDA receptors induces a glutamate surge either by a preferential inhibitory effect on fast-spiking interneurons (disinhibition hypothesis) or through direct effects on pyramidal neurons (direct hypothesis). This leads to activation of AMPA receptors and activates downstream factors related to plasticity, such as brain-derived neurotrophic factor (BDNF) and mTor (Miller et al. 2016). The effects of ketamine could be due to a mixture of the effects of the S and R enantiomers, which might have different mechanisms (Jelen et al. 2021, Wei, Chang et al. 2022).

DCS, on the other hand, acts as a partial agonist at the glycine site on the NR1 subunit of the NMDA receptor, meaning that it is expected to act in a state-dependent manner, i.e. as an agonist at low doses and as a functional antagonist of the NMDA receptor at higher doses. Indeed, our results indicate that at a dose of 1000 mg, the effects of DCS are similar to the pore-blocking antagonist ketamine, enhancing gamma, whereas at lower doses it does not. Recent preclinical work, however, found that ketamine increases the firing rate of medial prefrontal pyramidal cells without supressing the activity of fast-spiking interneurons in the mPFC (Amat-Foraster et al. 2018, Amat-Foraster, Celada et al. 2019). They hypothesised that inhibition of the activity of long-range GABAergic afferents or increased excitation from sub-cortical areas is responsible for the increase in firing of mPFC pyramid cells. Since gamma is often generated in a network of both pyramid cells and fast-spiking interneurons, and requires NMDA receptor activity (Carlen et al. 2012), the increase in firing rates of pyramidal cells without inhibition of fast spiking interneurons could be expected to increase gamma oscillations measured using EEG. There are at least two possible mechanisms by which HFO might be increased: firstly, the increased activity of pyramid neurons is likely to increase broadband activity which has an HFO component (Leszczynski, Barczak et al. 2020). This is supported by a recent study in mice (Guyon et al. 2021). However, in view of the rounded peak in the percentage change spectra above 100 Hz (see Fig. 2), this is unlikely to fully account for the HFO increase during the infusion. Secondly, increased activation of striato-cortical circuits can lead to increased HFO activity (Flores et al. 2015).

It has recently been shown that ketamine blocks burst firing in the lateral habenula, and improves despair and anhedonia as measured in rodents, which may be a mechanism of action of ketamine (Yang et al. 2018). The authors propose a model where ketamine disinhibits downstream monoaminergic reward centres. The bursts are in the 4–10-Hz theta band in rodents, which raises the question of whether the theta reduction we saw with ketamine, but not DCS (see Online Resources 2), was due to blockade of lateral habenula burst firing. Ketamine also reduces theta in the hippocampus (Lazarewicz et al. 2010a, b) and hippocampal theta synchronises with both lateral habenula (Aizawa et al. 2013) and medial prefrontal areas (Siapas et al. 2005). A reduction in theta might also have a knock-on effect in the higher frequencies. For example, frontal gamma and high-frequency oscillations can be phasically inhibited at theta frequencies (Zhong et al. 2017). Ketamine-associated theta modulation of high frequencies has been demonstrated with breathing-related theta in rats, although in humans breathing rate is well below theta frequencies (Zelano et al. 2016; Wróbel et al. 2020), and human breathing rate has not been found to be altered by ketamine. A release from pulsed inhibition at slow frequencies, such as theta, would be expected to produce an overall increase in amplitudes at higher frequencies. Alternatively, it could be speculated that a ketamine-induced dysfunction of theta oscillations in connections from the hippocampus/lateral habenula to striatal and medial prefrontal areas could destabilise beta oscillations in the striatal/prefrontal circuits, and thus produce a shift from beta to higher frequencies (Nottage et al. 2013; Brittain and Brown 2014).

### Ketamine and metabolites and EEG

Despite initial reports that the metabolite HNK has antidepressant properties in itself, more recent studies have cast doubt on this hypothesis (Abdallah 2020), and a recent clinical study found that higher HNK plasma levels after ketamine were associated with a weaker antidepressant effect (Farmer et al. 2020). Farmer and colleagues also found that in treatment-resistant depression, peak HNK levels were positively correlated with gamma, whilst norketamine levels were negatively correlated with gamma. We did not find such associations in our study. In fact, in our data, there was a trend towards a positive correlation between the mean percentage change from baseline at 40–65 Hz on the ketamine day and the mean plasma levels of norketamine. There are three potential reasons for this difference: firstly, they tested people with depression whilst our data was from healthy subjects; secondly, their EEG recordings were made 6–9 h after ketamine administration whilst ours were during the ketamine infusion and less than 3 h afterwards; and thirdly, the location of the EEG channels differed between studies.

Although not part of the original hypotheses, we did notice a strong negative correlation between (2S, 6S)-HNK during the ketamine infusion and absolute magnitude of alpha, not just in treatment EEG sessions, but also in baseline, pre-treatment magnitude and, possibly, a positive correlation with norketamine 2.5 h later. Hence, individuals who generally had high amplitude alpha oscillations tended to metabolise ketamine into (2S, 6S)-HNK less rapidly, leaving more residual norketamine. This suggests the intriguing possibility that individual differences in the magnitude of alpha might, in some way, be associated with individual differences in ketamine metabolism. Specifically, the activity of the enzymes CYP2A6 and CYP2B6, which catalyse this conversion of ketamine into HNK (Lavender et al. 2020), might be lower in people with more pronounced alpha oscillations. Farmer and colleagues reported lower levels of HNK acutely following ketamine, which corresponded to higher baseline alpha in our study, predicted lower depressive symptoms 3–7 days later (Farmer et al. 2020), consistent with the reports of high baseline alpha corresponding to good antidepressant response (Wade and Iosifescu 2016). It could be speculated that the reason for the discrepancy between Farmer’s results and the preclinical work indicating HNK has antidepressant properties might be that the association between high HNK and poor response to ketamine might not point to a direct effect of HNK, but rather to an individual’s ketamine metabolism being related to antidepressant response. It is well established that alpha power is highly heritable (Begleiter and Porjesz 2006), although developmental environment may also have an effect on the magnitude of frontal alpha (Zietsch et al. 2007). Furthermore, there are wide variations in HNK concentrations after ketamine administration, which are thought to be due to genetic variability in the relevant cytochrome P450s (Desta et al. 2012). The question arises as to whether there might be some association between these two different sets of genetic variability, or alternatively whether some latent environmental, metabolic or other variable is influencing both metabolism from ketamine to HNK and alpha power.

Individual differences in ketamine metabolism cannot account for the negative correlations we observed between beta and the maximum values of (2S, 6S)-HNK and (2R, 6R)-HNK as these associations are not in the baseline EEG and are present in the percentage change from placebo. Hence, frontal beta levels may be reduced via the activity of HNK, although this is not consistent with preclinical work (Zanos et al. 2019). Frontal beta is generated in fronto-striatal circuits, and a recent review reported increased beta in people with depression (Newson and Thiagarajan 2018). Beta in prefrontal areas is involved in supporting cognitive processes and is dependent on the mediodorsal thalamus (Parnaudeau et al. 2013). The role of prefrontal beta may be to actively maintain the current cognitive set (Engel and Fries 2010). The suppression of beta we observed may be linked to inhibition of MD thalamus by ketamine as shown in anaesthetised rats (Amat-Foraster et al. 2018) although reduced MD neuronal firing with ketamine was not observed in a more recent study in awake and freely moving rats (Amat-Foraster, Celada et al. 2019). Alternatively, beta reduction may result from inhibition of local PFC somatostatin interneurons by ketamine (Chen, Zhang et al. 2017, Ali et al. 2020), and reflect deficits in somatostatin interneurons in people with MDD (Fee et al. 2017), as a result of direct inhibition of cortical pyramid cells (Miller et al. 2016), or finally, as a knock-on effect of theta disruption, as discussed earlier.

### Psychomimetic changes

In terms of subjective changes, our predictions were met as ketamine increased scores on the PSI similar to previous studies. This effect was transient as the scores were no longer raised at the third timepoint, approx. 2–2.5 h after the ketamine infusion, which is the time window where antidepressant effects have been shown to emerge. As expected, the levels of ketamine metabolites persisted, or increased, after the infusion stopped, indicating that these metabolites do not appear to contribute to the psychotomimetic effects. DCS, even at the higher dose, did not significantly increase subjective psychotomimetic experiences, although there was a trend increase in perceptual distortion ratings, but its significance did not survive correction for multiple comparisons.

Our previous THC study showed that the ratio of posterior low gamma to anterior high beta was positively correlated with symptoms of reality distortion. In the current study, the ratio of posterior 27–40 Hz to anterior 20–27 Hz was positively correlated with perceptual distortion ratings at Time 2 (during ketamine infusion), which adds evidence to this ratio being a general biomarker for reality distortion. This has been hypothesised (Nottage et al. 2015) to be due to a relative increase in excitation in the cortical-pulvinar circuits (indexed by posterior low gamma (Halassa and Kastner 2017)) compared to a relatively lower level of activation of the fronto-striatal cognitive control circuits, as indexed by frontal high beta (Engel and Fries 2010; Parnaudeau et al. 2013; Sherman et al. 2016) with the end result that neural noise is not sufficiently controlled and is perceived as real stimuli. It is also of interest that the ketamine-induced changes in the perceptual distortion component of the PSI have previously been found to be positively correlated with the fMRI signal in a superior, midline area of the parietal cortex (Stone et al. 2015), which is beneath the parietal electrode location used in this study. Furthermore, recent preclinical data show that the antipsychotic clozapine reverses the ketamine-induced reduction in a 10–20-Hz range in rats, supporting the involvement of beta band activity in the psychotomimetic effects induced by ketamine (Bowman, Richter et al. 2022).

### EMG artefacts

We have been developing methods for dealing with artefacts in the gamma band and above for a number of years, and the optimisation and validation of these methods for frequencies above 100 Hz is important. However, the data presented in this article are from midline channels only, which are known to have minimal muscle contamination (Fz and Pz, referenced to Cz). There are no muscle fibres directly below these electrodes, and they are located some distance away from those muscles whose activity contaminates much of the EEG signal. Previous studies have confirmed that muscle artefacts in such midline channels are confined to periods of gross muscle contraction of the masseter, frontalis or neck muscles. These periods were excluded from the analysis. We are therefore confident that the signal-to-noise ratio in these dipolar channels is sufficiently high that the results can be relied upon without further correction.

### Limitations

Gender differences have been observed in studies of EEG and antidepressant response (Arns et al. 2016). One limitation of our study is that only male participants were tested, and so it is unknown if the results will apply to females.

The a priori focus of this study was HFO in medial frontal areas. We therefore opted to analyse only data from three midline channels which are least contaminated by EMG artefacts. However, ketamine and DCS may have produced different effects in other brain areas which were not part of our a priori analysis plan. For the lower frequencies, more complex methods such as source localisation would have yielded more information, but they would not have been suited to the analysis of HFO in the scalp EEG. Also, only analysing the eyes closed data could bias the results, and whilst we believe this to have been necessary to enable us to extract the high frequencies, eyes open data should ideally be included for studying the lower frequencies. We do not have sufficient eyes open data in this study for this analysis.

Whilst we interpret the effects of ketamine as mediated by the NMDA receptor pore block, arguably its most important pharmacological action, leading to higher glutamate levels which correlate with the psychotomimetic effects (Stone et al. 2012) ketamine is known to have effects at other receptors, including cholinergic, noradrenergic and dopaminergic (Sleigh et al. 2014), and their contribution to psychotomimetic and other effects we observed is not well understood.

## Conclusions and future work

This study has demonstrated that neural oscillations above 100 Hz, in the human scalp EEG, can be a useful biomarker in drug studies. Such oscillations are increasingly being reported in preclinical studies, and therefore have potential as a translatable biomarker. We also found that two different glutamatergic antidepressants enhanced the activity of frontal high-frequency oscillations, leading to the proposal that drug-related increases in HFOs might index antidepressant potential. On the other hand, ketamine increased the mid gamma oscillations to a much greater extent than DCS especially in a parietal location and also reduced frontal beta magnitude. The shift in activity from frontal high beta to posterior gamma may reflect ketamine’s psychomimetic effects. Further research is needed into the relationship between high-frequency oscillations and RAADs and psychosis-inducing agents to establish whether these effects generalise to other drugs. More methodological work is also needed to improve and validate methods for cleaning the high frequencies of the EEG, so that HFO in locations outside the midline can be utilised in pharmacological studies. Our results may be useful in the development of future RAADs, as they suggest that effective, psychomimetic free, RAADs may increase HFO in the frontal areas without increasing mid to low gamma in posterior areas or decreasing frontal high beta.

## Supplementary Information

Below is the link to the electronic supplementary material.

Supplementary file1 (PDF 92 KB)

Supplementary file2 (PDF 181 KB)

Supplementary file3 (PDF 150 KB)

Supplementary file4 (PDF 164 KB)
