# Supplementary figures and images for: The effect of ketamine and D-cycloserine on the high frequency resting EEG spectrum in humans
Source: Psychopharmacology (Berl). 2022 Nov 19;240(1):59–75. doi: 10.1007/s00213-022-06272-9 (PMC9816261; doi:10.1007/s00213-022-06272-9)

## Online Resources 1 – Protocol Flowchart

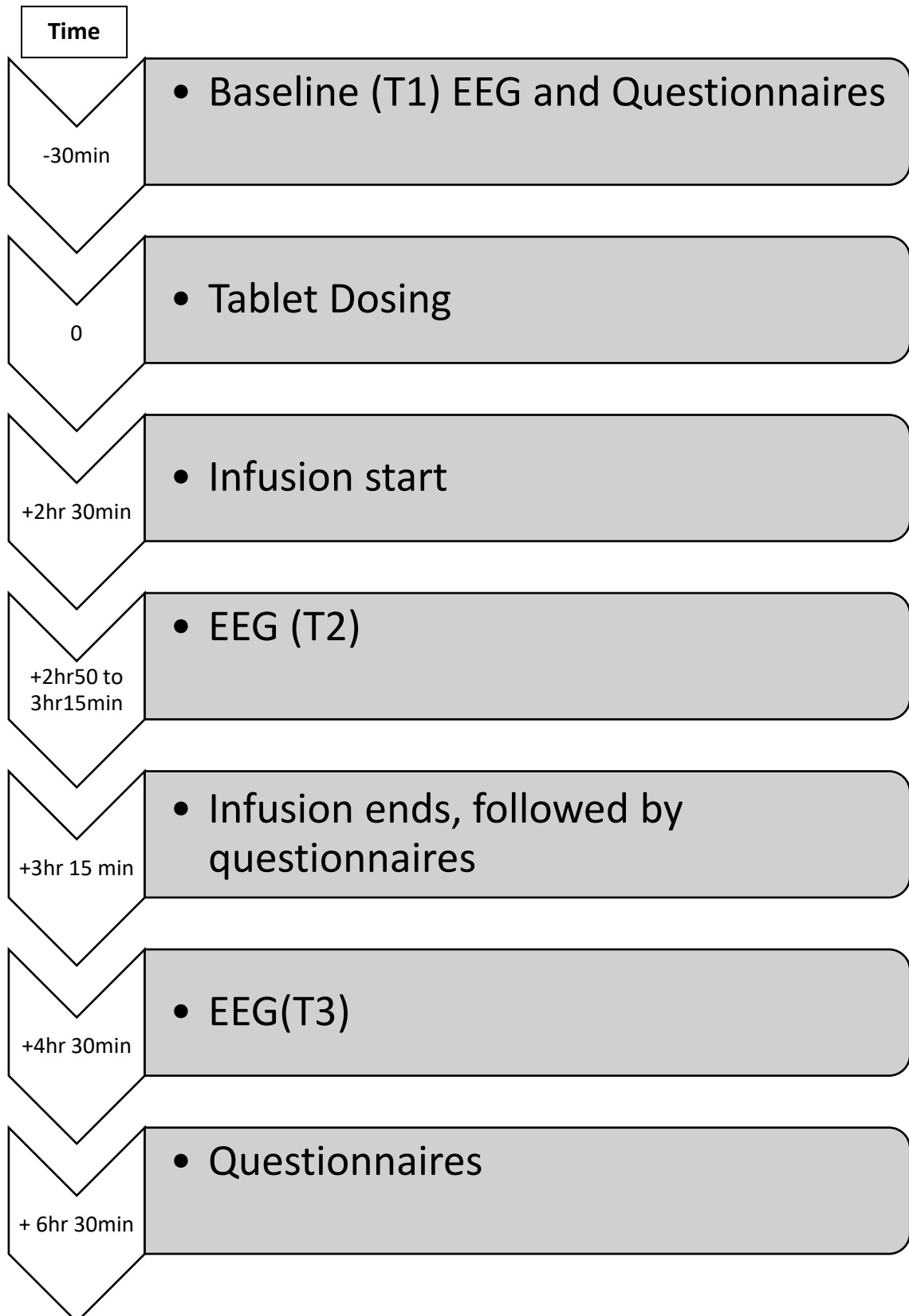

Supplement: Supplementary file 1 — Supplementary file1 (PDF 92 KB) [file 213_2022_6272_MOESM1_ESM.pdf]
