# Supplementary material for: The effect of ketamine and D-cycloserine on the high frequency resting EEG spectrum in humans
Source: Psychopharmacology (Berl). 2022 Nov 19;240(1):59–75. doi: 10.1007/s00213-022-06272-9 (PMC9816261; doi:10.1007/s00213-022-06272-9)
Supplement: Supplementary file 2 — Supplementary file2 (PDF 181 KB) [file 213_2022_6272_MOESM2_ESM.pdf]

Online Resources 2 – The Magnitude of resting oscillations below 20Hz at Time 2 (peak of ketamine) compared with placebo

EEG analysis

The amplitudes of frequencies below 20Hz were also calculated in the same Fz-Cz and Pz-Cz bipolar derivations that were used for the high frequency analysis. The initial stages of the EEG analysis are the same as for the high frequencies. The magnitudes of the low frequencies were extracted using moving-window, short-time Fourier transforms with a Hann window, applied to the eyes-closed data. Four bands were extracted: Theta (4-8Hz), Low Alpha (8-10Hz) High Alpha (10-13Hz) and Low beta (13-20Hz). For the Fourier Transforms, Theta and Alpha were quantified using 1 sec windows, and beta using 0.5 sec windows, with a 2.5% shift between consecutive windows.

It should be noted that we have not corrected for ocular artifacts. However, only the eyes-closed data was analysed, and in the closed eyes closed condition there are sometimes slow rolling horizontal eye movements, but not blinks or saccades. The rolling horizontal eye movements primarily affect the delta band at locations frontal locations lateral to the eyes. As we only analysed the midline signals down to 4Hz, we do not believe that our results could have been affected by ocular artifacts.

Statistics

Log transformed values were analyzed using IBM SPSS. For each drug and measure separately, a mixed models analysis was carried out at Time 2 (Peak of ketamine). Condition was included as a fixed effect, with 3 levels for the DCS analysis (1000mg, 250mg and Placebo) and two levels for the Ketamine analysis (Drug and Placebo). Day was included as a covariate, whilst Subject was included as a random effect.

Results

These results have not been corrected for multiple comparisons.

Table ES1 The effect of ketamine, vs placebo, on frontal frequencies below 20Hz

Frontal	*Theta (4-8 Hz)*	*Low Alpha (8-10Hz)*	*High Alpha (10-13Hz Hz)*	*Low Beta (13-20Hz)*
F	12.25	8.39	2.37	50.13
*p**	0.0021	0.008	0.14	$4x10^{-7}$
df	28.6	21.7	22.0	21.9
Cohen's d	-0.50	-0.39	-0.27	-1.20
Ketamine (µV/Hz)	3.094	1.699	1.775	1.411
Placebo (µV/Hz)	3.740	2.046	2.024	2.018

Table ES2 The effect of ketamine, vs placebo, on parietal frequencies below 20Hz

	Parietal	*Theta (20-27 Hz)*	*Low Alpha (8-10Hz)*	*High Alpha (10-13Hz Hz)*	*Low Beta (13-20Hz)*
	F	20.33	5.43	0.18	11.83
	*p**	0.00018	0.03	0.67	0.002
	df	28.5	21.8	22.0	21.7
	Cohen's d	-0.64	-.30	0.07	-0.47
	Ketamine (µV/Hz)	3.121	0.901	3.025	1.982
	Placebo (µV/Hz)	4.080	1.086	2.907	2.356

Also, 1000mg DCS reduced frontal low beta (p=0.006) and parietal high (p=0.008) and low alpha (p=0.001) compared with placebo, although these effects would not survive correction for multiple comparisons.

Conclusions

Both ketamine and the high dose of DCS reduced the magnitude of frontal low beta magnitude, but this effect was much more significant with ketamine. Whereas ketamine also reduced theta magnitude, 1000mg DCS tended to reduce alpha but left theta unchanged (p=0.49).
