# Supplementary material for: The effect of ketamine and D-cycloserine on the high frequency resting EEG spectrum in humans
Source: Psychopharmacology (Berl). 2022 Nov 19;240(1):59–75. doi: 10.1007/s00213-022-06272-9 (PMC9816261; doi:10.1007/s00213-022-06272-9)
Supplement: Supplementary file 3 — Supplementary file3 (PDF 150 KB) [file 213_2022_6272_MOESM3_ESM.pdf]

Online Resources 3 - Plasma levels and EEG

Table ES3 Correlations of percentage change in high frequency oscillations at Peak (T2) vs plasma levels of ketamine and metabolites (Mean of during and post infusion)

Percentage Change From Placebo		Ketamine	Norketamine	HNK
Frontal low gamma (27-40Hz)	Pearson Correlation	.020	.254	-.006
	Sig (2-tailed)	.928	.242	.980
	N	23	23	19
Parietal low gamma (27-40 Hz)	Pearson Correlation	-.127	.176	-.023
	Sig (2-tailed)	.564	.423	.925
	N	23	23	19
Frontal Mid Gamma (40-65 Hz)	Pearson Correlation	.217	**.436**	-.074
	Sig (2-tailed)	.321	**.038**	.763
	N	23	**23**	19
Parietal Mid Gamma (40-65 Hz)	Pearson Correlation	-.005	.282	.002
	Sig (2-tailed)	.982	.192	.993
	N	23	23	19
Frontal High Gamma (65-100 Hz)	Pearson Correlation	-.034	.116	-.166
	Sig (2-tailed)	.879	.598	.498
	N	23	23	19
Parietal High Gamma (65-100Hz)	Pearson Correlation	-.194	.105	-.161
	Sig (2-tailed)	.375	.635	.510
	N	23	23	19
Frontal HFO (100-170 Hz)	Pearson Correlation	-.191	-.073	-.188
	Sig (2-tailed)	.381	.742	.441
	N	23	23	19
Parietal HFO (100-170Hz)	Pearson Correlation	-.378	-.093	-.317
	Sig (2-tailed)	.076	.673	.187
	N	23	23	19

Note: The correlations which are significant without correction for multiple comparisons are shown in bold.

Table ES4: Correlations of the Magnitude of oscillations below 27Hz at Peak (T2) vs plasma levels of ketamine and metabolites

Peak (T2) Log transformed Amplitude (Fz-Cz)		Keta-mine (During Infusion)	Nor-ketamine (During infusion)	(2S,6S)-HNK (During Infusion)	Keta-mine (Post infusion)	Nor-ketamine (Post infusion)	(2S, 6S)-HNK (Post infusion)	(2R, 6R)-HNK (Post infusion)
Theta 4-8Hz	Pearson Correlation	-.146	.138	-.411	-.215	.318	-.151	-.229
	Sig (2-tailed)	.496	.520	.072	.325	.140	.492	.292
	N	24	24	20	23	23	23	23
Low Alpha 8-10Hz	Pearson Correlation	-.160	.104	**-.546**	-.306	.313	-.383	-.403
	Sig (2-tailed)	.455	.628	**.013**	.155	.146	.071	.056
	N	24	24	**20**	23	23	23	23
High Alpha 10-13Hz	Pearson Correlation	-.211	.095	**-.652**	**-.456**	.288	**-.500**	**-.508**
	Sig (2-tailed)	.322	.660	**.002**	**.029**	.182	**.015**	**.013**
	N	24	24	**20**	**23**	23	**23**	**23**
Low Beta (13-20 Hz)	Pearson Correlation	-.193	.159	**-.671**	-.368	**.430**	**-.475**	**-.540**
	Sig (2-tailed)	.366	.458	**.001**	.084	**.041**	**.022**	**.008**
	N	24	24	**20**	23	**23**	**23**	**23**
High Beta (20-27 Hz)	Pearson Correlation	-.190	.172	**-.631**	-.383	**.486**	**-.533**	**-.557**
	Sig (2-tailed)	.373	.421	**.003**	.071	**.019**	**.009**	**.006**
	N	24	24	**20**	23	23	**23**	**23**

Note: The correlations which are significant without correction for multiple comparisons are shown in bold. (2S, 6S)-HNK (Post infusion) is omitted because there is insufficient data.
