# Supplementary material for: The effect of ketamine and D-cycloserine on the high frequency resting EEG spectrum in humans
Source: Psychopharmacology (Berl). 2022 Nov 19;240(1):59–75. doi: 10.1007/s00213-022-06272-9 (PMC9816261; doi:10.1007/s00213-022-06272-9)
Supplement: Supplementary file 4 — Supplementary file4 (PDF 164 KB) [file 213_2022_6272_MOESM4_ESM.pdf]

Online Resources 4. Details of EEG pre-processing using GammArt software

i. Filtering and rejection of noisy segments

A high pass Finite Impulse Response (FIR) was generated using the *designfilt* function in MATLAB, with the filter order set as 5000 and the cut-off set as 1 Hz. This was applied to the data using the MATLAB *filtfilt* function.

Periods of gross, widespread EMG artifacts affecting multiple channels were identified with a method adapted from Fieldtrip's well-established 'ft_artifact_muscle' function (Oostenveld et al. 2011). EEG data for 14 peripheral channels which can be strongly affected by this kind of noise (FP1,FP2,F7,F8,FT7,FT8,T7,T8,TP7,TP8,P7,P8,O1,O2) was bandpass filtered at [110,140]Hz, using a FIR filter with order 1000. High amplitudes in this frequency band are a good indicator of gross and widespread EMG artifacts. A channel's instantaneous amplitudes of uncontaminated scalp EEG in this frequency band are roughly normally-distributed, which means that the gross EMG artifacts can be identified as outliers to this distribution, with amplitudes often 10-100 times larger than the uncontaminated amplitudes. The instantaneous amplitude of the bandpass-filtered signal was determined using the Hilbert transform. Each channels' amplitude values were then transformed to the canonical normal distribution using a robust z-transform (using median and median absolute deviation to perform the z transform). Data points with a mean absolute z-value, averaged over all 14 peripheral channels, of more than 5 were identified as outliers and those data points were excluded from the subsequent frequency analysis of (FZ-CZ) and (FZ-PZ).

ii. Power-line correction using noise cancellation

One electrode was used to record the power-line signal. The signal from this electrode was band pass filtered with a FIR filter, generated using the designfilt function in MATLAB. The filter order was set as 5000 and the high and low cut-off frequencies were 48 Hz and 52Hz, respectively. Fluctuations in the amplitude of the power-line channel were adjusted for by normalising the signal. A cosine wave was generated by phase shifting the sine wave by $\pi/2$ and harmonics of the power-line signal were generated using the trigonometric relationship: $\sin(A+B) = \sin A \cos B + \cos A \sin B$

A weighted least square, moving variance, method was used to determine the regression weights for the power-line signal and the harmonics. This was achieved using the MATLAB *movvar* function, with a window length of 0.2 seconds. The power-line signal and harmonics were subsequently subtracted from the data.

iii Fast Fourier Transforms

The power spectral density was calculated using the MATLAB *pwelch* function. The window was calculated using the MATLAB *hann* function, with the *periodic* option. The amplitude values were obtained from the square-root of the power values. HFO were quantified using 100ms windows, gamma using 200ms windows and beta using 500ms windows, with a 2.5% shift between consecutive windows.
